# Enhancing Photovoltaic Performance of GaAs Single-Junction Solar Cells by Applying a Spectral Conversion Layer Containing Eu-Doped and Yb/Er-Doped Phosphors

**DOI:** 10.3390/nano9111518

**Published:** 2019-10-25

**Authors:** Wen-Jeng Ho, Jheng-Jie Liu, Zong-Xian Lin, Hung-Pin Shiao

**Affiliations:** 1 Department of Electro-Optical Engineering, National Taipei University of Technology, No. 1, Section 3, Zhongxial East Road, Taipei 10608, Taiwan; jjliu@mail.ntut.edu.tw (J.-J.L.); d0933463284@gmail.com (Z.-X.L.); 2 Win Semiconductor Corp., Taoyuan 333, Taiwan; hpxiao@winfoundry.com

**Keywords:** europium-doped (Eu-doped), GaAs solar cell, luminescent downshift, phosphors, up-conversion, ytterbium/erbium-doped (Yb/Er-doped)

## Abstract

In this study, we examined efforts to increase the photovoltaic performance of GaAs single-junction solar cells using spectral conversion layers, respectively, composed of europium-doped (Eu-doped) phosphors, ytterbium/erbium-doped (Yb/Er-doped) phosphors, and a combination of Eu-doped and Yb/Er-doped phosphors. Spin-on film deposition was used to apply the conversion layers, all of which had a total phosphor concentration of 3 wt%. The chemical compositions of the phosphors were examined by energy-dispersive X-ray spectroscopy. The fluorescence emissions of the phosphors were confirmed by using photoluminescence measurements. Under laser diode excitation at 405 nm, we observed green luminescent downshift (LDS) emissions by Eu-doped phosphors at wavelengths of 479 nm to 557 nm, and under excitation at 980 nm, we observed red up-conversion (UC) emissions by Yb/Er-doped phosphors at wavelengths of 647 nm to 672 nm. The spectral conversion layers were characterized in terms of optical reflectance, external quantum efficiency, and photovoltaic current and voltage under AM 1.5 G simulations. The conversion efficiency of the cell combining Eu-doped and Yb/Er-doped phosphors (23.84%) exceeded that of the cell coated with Yb/Er-doped phosphors (23.72%), the cell coated with Eu-doped phosphors (23.19%), and the cell coated without phosphors (22.91%).

## 1. Introduction

Single-junction solar cells only convert photons with energy equal to and above the bandgap of the active material in the solar cells. Photons carrying energy below the bandgap (Eg) are not absorbed at all, i.e., their energy is not used for the generation of photocarriers. Almost all photons with energy exceeding the bandgap are absorbed, however, the excess energy (>>Eg) is lost due to thermalization of the photocarriers they generate. The spectral losses of single-junction solar cells (e.g., silicon and GaAs) limit the theoretical photovoltaic efficiency to about 30% to 33% [1,2,3]. The mismatch between the incident solar energy spectrum and the spectral absorption of solar cell is a crucial issue [4,5,6]. Considerable effort has gone into the creation of high-efficiency single-junction solar cells by overcoming the conversion efficiency limit. This has led to the development of multiple bandgap tandem cells [7,8,9,10,11], intermediate bandgap cells [12,13,14], down-conversion (DC) cells [15,16,17,18,19,20], up-conversion (UC) cells [21,22,23,24,25,26], and luminescent downshifting (LDS) cells [27,28,29,30]. In recent years, luminescent-phosphors materials for DC, UC, and LDS, which are capable of converting a broad spectrum of light into photons of particular wavelengths, have been synthesized and used to minimize the losses in the photovoltaic device energy conversion process. The spectral conversion layer of DC and LDS cells is generally located on the top surface of the cell, as it is meant to convert ultraviolent (UV) photons (E >> Eg) into visible (green-rich, red-rich) photons. The spectral conversion layer of UC cells is generally located on the bottom side of solar cells, as it is meant to modify photons that are not absorbed by the solar cell by shifting IR (infrared) and NIR (near infrared) parts of the spectrum to the visible part. Extensive research has been done to study spectral conversion on silicon-based solar cells, which has involved applying a DC-layer, or LDS-layer, to the front side of silicon-based solar cells, however, few researchers have applied a UC-layer to the front side of GaAs single-junction solar cells [31,32,33,34,35,36]. Furthermore, few researchers have simultaneously applied LDS-phosphors and UC-phosphors on the front side to enhance the conversion efficiency of GaAs single-junction solar cells.

In this study, we sought to enhance the photovoltaic performance of GaAs single-junction solar cells by applying spectral conversion layers, respectively, composed of europium-doped (Eu-doped) phosphors, ytterbium/erbium-doped (Yb/Er-doped) phosphors, and a combination of Eu-doped and Yb/Er-doped phosphors. We employed spin-on-film technique in the application of LDS- and UC-phosphors solution to the front side of GaAs solar cells and then examined the optical and electrical properties in terms of photoluminescence (PL), optical reflectance, and external quantum efficiency (EQE). We then confirmed the degree to which these efforts enhanced photovoltaic performance using photovoltaic current density and voltage (J-V) measurements. Finally, we compared the efficacy of LDS- and UC-phosphors for enhancing conversion efficiency of GaAs single-junction solar cells.

## 2. Experimental Details

### 2.1. Preparation and Characterization of Luminescent Downshifting Eu-Doped Phosphor Layer and Up-Conversion Yb/Er-Doped Phosphor Layer

Spin-on deposition was used to coat clean silicon substrate with a SiO_2_ layer containing Eu-doped silicate-phosphors or Yb/Er-doped yttrium oxide (Y_2_O_3_) phosphors at a concentration of 3 wt%. The coating solution was composed of 1.94 g of Silicafilm-5000 solution (Emulsitone Company product, Whippany, NJ, USA) mixed with 0.06 g of Eu-doped silicate-phosphors powder or 0.06 g Yb/Er-doped Y_2_O_3_-phosphors powder. Spin-on deposition was performed at 3000 rpm for 60 s before baking the substrates at 200 °C for 30 min under an air atmosphere. The thickness of a SiO_2_ layer containing Eu-doped or Yb/Er-doped phosphors was non-uniformly about 85 nm to 100 nm, which was dependence on the spacing and coverage of particles. For comparing, we also prepared samples with a 94 nm thick layer of pure SiO_2_ (using the same Silicafilm-5000) without any phosphor particles. The chemical compositions of the samples with proposed phosphors were examined using energy-dispersive X-ray spectroscopy (EDS) (JSM-6500, JEOL Ltd., Tokyo, Japan), and the fluorescence emissions were confirmed by photoluminescence measurements (Ramboss 500i Micro-PL Spestroscopy, DONGWOO Optron, Korea) using light sources with wavelengths of 405 nm for characterizing Eu-doped phosphors and 980 nm for characterizing Yb/Er-doped phosphors, respectively.

### 2.2. Growth of Epitaxial Layer and Fabrication of GaAs Single-Junction Solar Cells

**Figure 1:**
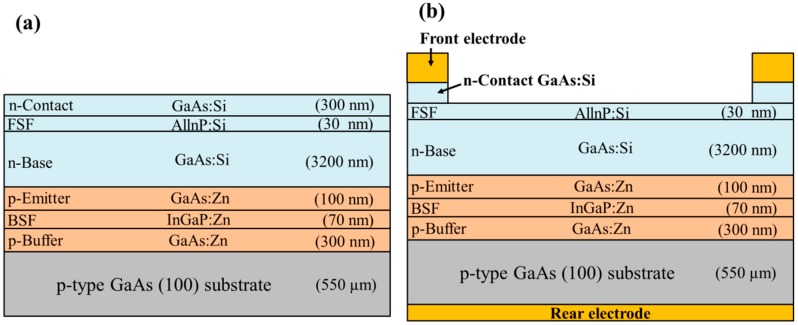
Schematic diagrams of (**a**) epitaxial layers in GaAs single-junction solar cell and (**b**) bare-type GaAs single-junction solar cell.

As shown in Figure 1a, the epitaxial layer structure in the GaAs single-junction solar cell was grown using metal-organic chemical vapor deposition (MOCVD) on a p^+^-GaAs (100) substrate. Arsine (AsH_3_) and phosphine (PH_3_) were used as group-V source gases, whereas trimethyl-Gallium (TMG), trimethyl-Indium (TMI), and trimethyl-Aluminum (TMA) were used as group III precursors. Disilane and dimethyl-zinc (DEZ) were used for n- and p-type doping, respectively. Growth was conducted over a temperature range of 635 to 650 °C under a chamber pressure of 15 mbar. First, a 300 nm thick p-GaAs (5 × 10^17^ cm^−3^) buffer layer was grown on the p^+^-GaAs substrate. This was followed by a 70 nm thick p-InGaP (5 × 10^17^ cm^−3^) back surface field (BSF) layer, a 100 nm thick p-GaAs (5 × 10^17^ cm^−3^) emitter layer, a 3200 nm thick n-GaAs (1 × 10^15^ cm^−3^) base layer, a 30 nm thick n-AlInP (5 × 10^17^ cm^−3^) front surface field (FSF) layer, and a 300 nm thick n^+^-GaAs (5 × 10^18^ cm^−3^) contact layer. The quality of the epitaxial layers was confirmed using double crystal X-ray diffraction, photoluminescence, scanning electron microscopy, and electrochemical capacitance-voltage measurements. The epitaxial layer structure of the proposed GaAs single-junction solar cell was grown using MOCVD and evaluated prior to device processing by a commercial epi foundry, WIN Semiconductors Corp.

The fabrication of GaAs single-junction solar cells involved depositing AuGe/Ni/Au grid-pattern n-contact electrodes on the n^+^-GaAs contact layer, as well as an AuBe/Ti/Au full-plane p-contact electrode on the rear surface of the p^+^-GaAs substrate via photolithograph lift-off processing and e-beam evaporation. The samples were then annealed at 385 °C for 300 s under ambient N_2_ to produce good ohmic contacts. Citric acid-solution was used for the selective removal of the n^+^-GaAs layer to expose the n-AlInP FSF layer, followed by mesa etching to isolate cells (referred to as a bare-type GaAs solar cell). The area of a bare-type GaAs solar cell was 1 cm^2^, as shown in Figure 1b.

**Figure 2:**
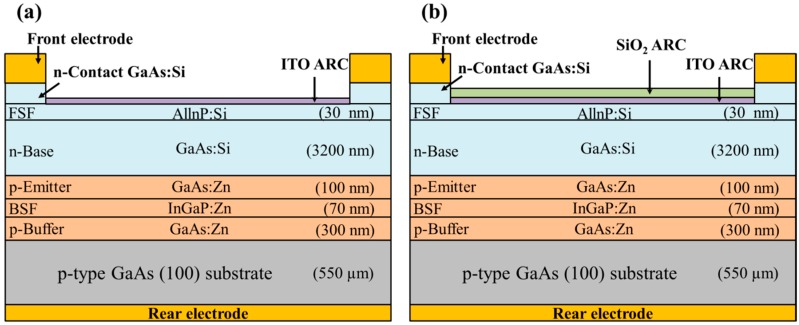
Schematic diagrams of bare-type GaAs single-junction solar cells coated with antireflection layers of (**a**) ITO and (**b**) ITO/SiO_2_.

We also evaluated the passivation and antireflection characteristics of indium tin-oxide (ITO) film and ITO/SiO_2_ films on the GaAs single-junction solar cells. Comparisons of electrical and optical performance involved preparing the following samples: the bare-type GaAs solar cell coated with a quarter-wavelength-thick ITO layer (74 nm) (Figure 2a) and the bare-type GaAs solar cell coated with a quarter-wavelength-thick ITO layer (74 nm) and, subsequently, with a quarter-wavelength-thick SiO_2_ layer (94 nm) (Figure 2b). The SiO_2_ film was deposited via spin-on film (because phosphor powder easy mixed into silicate solution to obtain a spectral conversion layer for following study), whereas the ITO film was deposited via RF (13.56 MHz) sputtering at a deposition rate of 3.84 nm/min with a substrate temperature of 255 °C and RF power of 45 W. A metallic In/Sn target (90:10 wt%, 5 cm in diameter) with purity of 99.99% was used as a source of ITO. The conductivity and optical transmittance of the deposited ITO film were examined using a four-point probe source meter (Keithley 2400, Keithley Instruments, Inc., Solon, OH, USA) and UV/VIS/NIR spectrometer (PerkinElmer Lambda 35, Waltham, MA, USA), respectively.

### 2.3. Fabrication and Characterization of GaAs Single-Junction Solar Cells Coated with SiO_2_ Layer Contained Eu-Doped Phosphors, Yb/Er-Doped Phosphors, and a Combination of Eu-Doped and Yb/Er-Doped Phosphors

**Figure 3:**
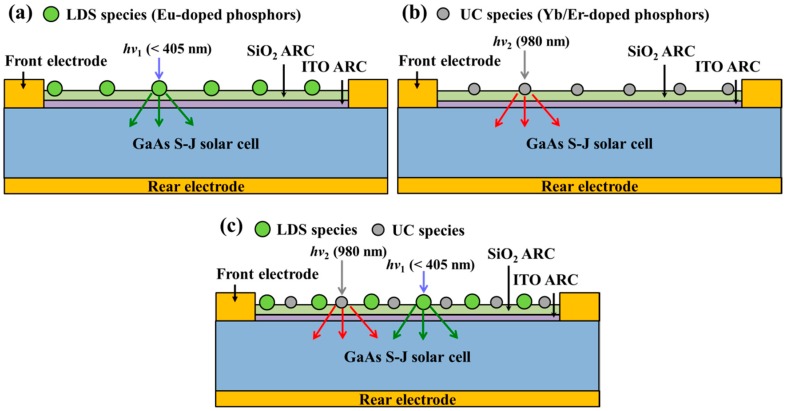
Schematic diagrams of GaAs single-junction solar cells coated with (**a**) Eu-doped phosphors in SiO_2_, (**b**) Yb/Er-doped phosphors in SiO_2_, and (**c**) a combination of Eu-doped and Yb/Er-doped phosphors in SiO_2_.

We investigated the degree to which conversion efficiency of GaAs single-junction solar cells could be enhanced by applying a SiO_2_ layer comprised of Eu-doped phosphors, Yb/Er-doped phosphors, or a combination of Eu-doped and Yb/Er-doped phosphors, in which the SiO_2_ layer was deposited on the ITO layer. This analysis was performed using the following samples: (a) GaAs solar cell coating with a layer of SiO_2_ that included 3 wt% Eu-doped phosphors, (b) GaAs solar cell coating with a layer of SiO_2_ that included 3 wt% Yb/Er-doped phosphors, and (c) GaAs solar cell coating with a layer of SiO_2_ that included a combination of Eu-doped phosphors (1.5 wt%) and Yb/Er-doped phosphors (1.5 wt%), as shown in Figure 3. Note that the total concentration of phosphors in the spectral conversion layer of all samples was 3 wt% for the sake of comparison. The mixtures of SiO_2_ solution (refer to Section 2.1) were applied to the GaAs solar cells with an ITO ARC via spin-on-film technique at 3000 rpm for 60 s before being baked at 200 °C for 30 min under air atmosphere. The thickness of the SiO_2_ layer in all samples was controlled to approximately 94 nm.

Reflectance spectra were collected using a miniature spectrometer (USB 4000, Ocean Optics, Inc., Largo, FL, USA) with a deuterium tungsten light source (200 nm to 2000 nm) and a reflective integrating sphere (diameter of 5 cm). The reduction in reflectance showing on the reflectance spectrum revealed the absorption band of LDS and UC phosphors materials. The EQE (Enli Technology Co., Ltd., Kaohsiung, Taiwan) response at wavelengths from 300 nm to 1100 nm was used to examine the spectral conversions of LDS and UC. Photovoltaic J-V measurements under 1 sun air mass (AM) 1.5 G simulations (1000 mW/cm^2^ at 25 °C) were used to confirm the contributions of the proposed spectral-conversion layers. The solar simulator (XES-151S, San-Ei Electric Co., Ltd., Osaka, Japan) was calibrated using a crystalline-silicon reference cell (PVM-894, PV Measurements Inc., Boulder, CO, USA) certified by the National Renewable Energy Laboratory (NREL) prior to the measurements of GaAs single-junction solar cells.

## 3. Results and Discussion

### 3.1. Material Characteristics of Luminescent Downshifting Eu-Doped Phosphors and Up-Conversion Yb/Er-Doped Phosphors

**Figure 4:**
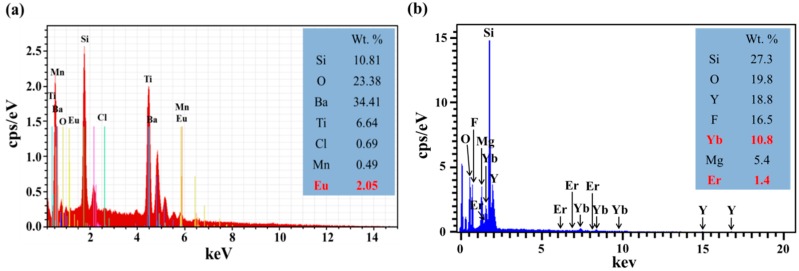
EDS spectra from silicon substrate coated with layer of SiO_2_ containing (**a**) Eu-doped silicate phosphors and (**b**) Yb/Er-doped Y_2_O_3_ phosphors.

EDS can be used effectively for element analysis. Each element within a sample possesses a unique atomic structure, which exhibits a unique set of peaks in its electromagnetic emission spectrum. Figure 4a shows the EDS spectrum of a silicon substrate coated with a SiO_2_ layer containing Eu-doped silicate phosphors composed primarily of Ba, O, Si, and Ti with small quantities of Eu, Cl, and Mn. Figure 4b shows the EDS spectrum of a silicon substrate coated with a SiO_2_ layer containing Yb/Er-doped Y_2_O_3_ phosphors composed primarily of Si, O, Y, and F with small quantities of Yb, Mg, and Er. The Y_2_O_3_-material features a high melting point, wide bandgap, high solubility between Yb^3+^ and Er^3+^, and good transparency at ultraviolet and infrared wavelengths.

**Figure 5:**
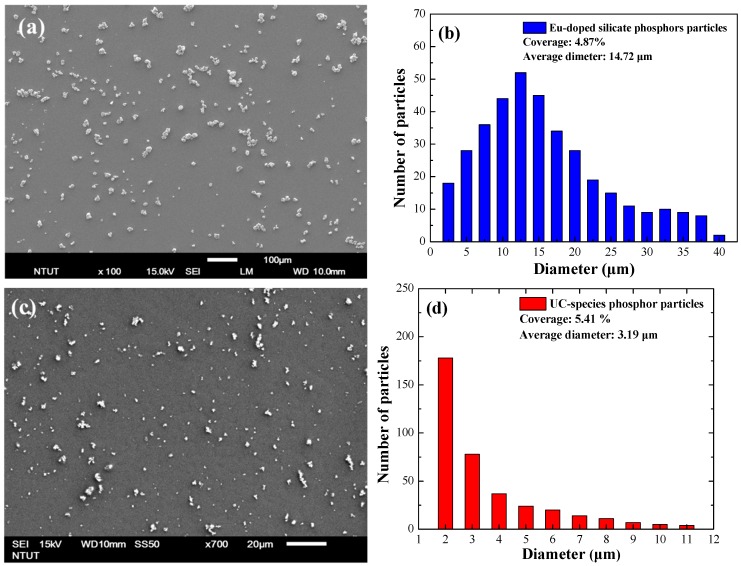
(**a**,**c**) Top view SEM images and (**b**,**d**) surface profiles of the samples with Eu-doped phosphors particles and Yb/Er-doped phosphors particles at concentration of 3 wt%, respectively.

**Figure 6:**
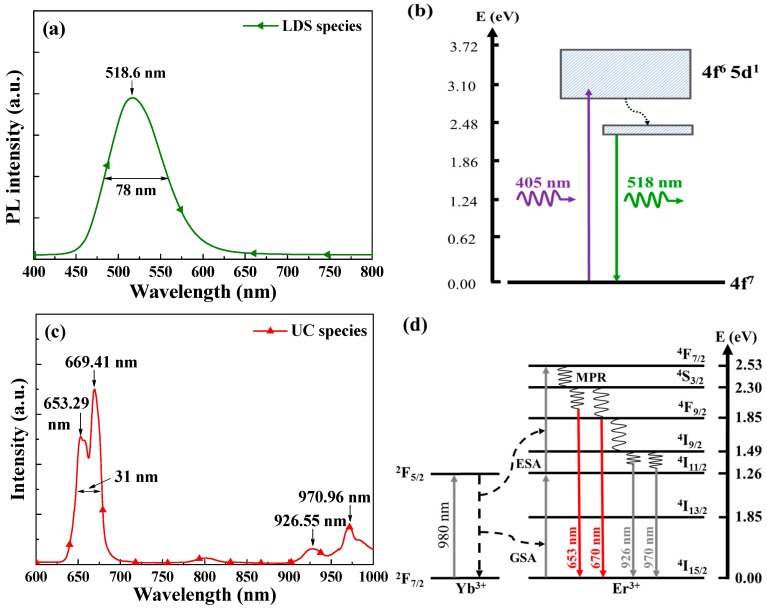
(**a**,**c**) PL fluorescence emission spectrum and (**b**,**d**) the energy level scheme from silicon substrate coated with a SiO_2_ layer containing of Eu-doped silicate phosphors under excitation at a wavelength of 405 nm and Yb/Er-doped phosphors under excitation at a wavelength of 980 nm, respectively.

Figure 5a–d presents top view SEM images and surface profiles of the samples with Eu-doped phosphors particles and Yb/Er-doped phosphors particles at a concentration of 3 wt%, respectively. The size distribution and coverage were calculated using Image-J software from the corresponding SEM images. The particle had an average diameter of approximately 14.72 μm and coverage of approximately 4.87% for the sample with Eu-doped phosphors, however, that of approximately 3.18 μm and coverage of approximately 5.41% for the sample with Yb/Er-doped phosphors. These particles would be scattered incident light, as well as had some influence on optical reflectance and EQE performance. Figure 6a presents the PL fluorescence emission spectrum from a silicon substrate coated with a SiO_2_ layer containing Eu-doped silicate-phosphors under direct excitation from the ^4^f_7_ level into the ^5^d_1_ level under radiation at 405 nm from a laser diode. The PL emission peak was at 518.6 nm and the full width at half maximum (FWHM) was approximately 78 nm. Figure 6b presents the energy level scheme of Eu^2+^ ions under excitation at a wavelength of 405 nm. The PL results indicate that incident photons with a wavelength of less than 405 nm were absorbed by the Eu-doped phosphors and re-emitted photons at visible wavelengths (480 nm to 560 nm), i.e., green luminescent downshift (LDS) behavior. Figure 6c presents the PL fluorescence emission spectrum of a silicon substrate coated with a SiO_2_ layer containing Yb/Er-doped Y_2_O_3_ phosphors under radiation at 980 nm from a laser diode. PL emission peaks showed at wavelengths of 653.29, 669.4, 926.6, and 970.9 nm. Strong PL signals were observed between 650 nm and 670 nm. Figure 6d presents the energy level scheme of Yb^3+^, Er^3+^ ions under excitation by a 980 nm laser diode. The PL results indicate that incident photons with a wavelength of 980 nm were absorbed by Yb^3+^ and Er^3+^ ions and then re-emitted photons at visible wavelengths (630 nm to 680 nm), i.e., red up-conversion (UC) behavior. The photons from LDS and UC were coupled into and absorbed within the active region of the GaAs solar cell to generate additional photocarriers, and thereby enhance cell efficiency.

### 3.2. Optical and Electrical Performance of GaAs Single-Junction Solar Cells Coated with an Antireflection Layer of ITO or ITO/SiO_2_

**Figure 7:**
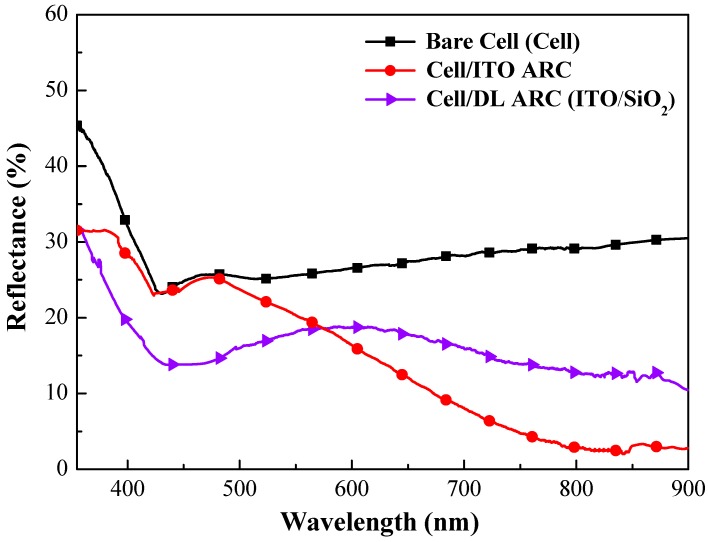
Optical reflectance spectra of bare-type GaAs single-junction solar cells and GaAs solar cells coated with layer of ITO or ITO/SiO_2_.

Figure 7 presents the optical reflectance spectra of bare-type GaAs single-junction solar cells and GaAs solar cells coated with an antireflection layer of ITO or ITO and SiO_2_ (refer to Figure 2a,b). Table 1 lists the weighted reflectance (*R_w_*) calculated over a wavelength range of 350 nm to 900 nm. For the sake of clarity, the *R_W_* and *EQE_W_* values of proposed cells were calculated as follows:

(1) $$R_{W}=\frac{\int_{350\;nm}^{900\;nm}R\left(\lambda \right)\phi_{ph}\left(\lambda \right)d\lambda }{\int_{350\;nm}^{900\;nm}\phi_{ph}\left(\lambda \right)d\lambda }\times 100\%$$

where *R*(*λ*) is the reflectance at a given wavelength (*λ*) and *ϕ_ph_*(*λ*) is the photon flux of AM 1.5 G at that wavelength (*λ*). The GaAs solar cell coated with an ITO layer displayed good antireflection performance with the lowest reflectance at 830 nm due to destructive interference. The ITO/SiO_2_ layers outperformed the ITO layer at wavelengths between 350 nm and 550 nm, however, it failed to match the performance of the ITO layer at wavelengths beyond 580 nm. The GaAs solar cells generally present higher responsivity values at longer wavelengths, resulting in the generation of more photocarriers.

**Figure 8:**
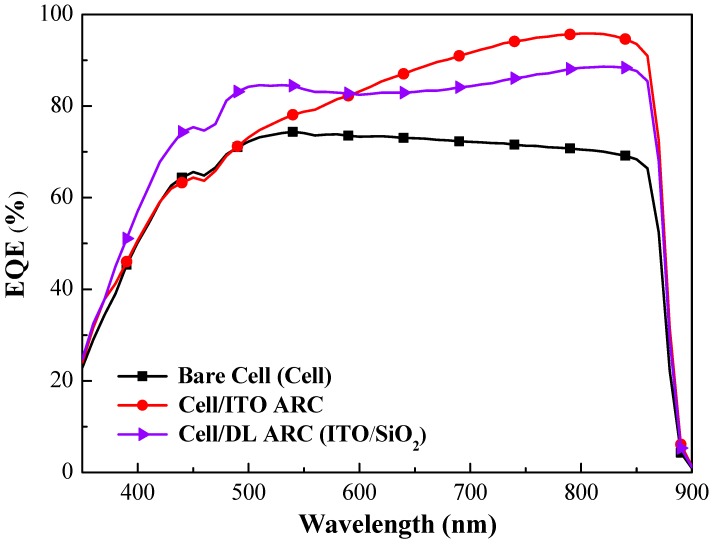
EQE response of bare-type GaAs single-junction solar cells and GaAs solar cells coated with layer of ITO or ITO/SiO_2_.

Figure 8 presents EQE response of bare-type GaAs single-junction solar cells and GaAs solar cells coated with a layer of ITO or layers of ITO/SiO_2_. Table 1 lists the weighted EQE (*EQE_w_*) of all cells calculated at wavelengths from 350 nm to 900 nm. For the sake of clarity, the *R_W_* and *EQE_W_* values of proposed cells were calculated as follows:

(2) $$EQE_{W}=\frac{\int_{350\;nm}^{900\;nm}EQE\left(\lambda \right)\phi_{ph}\left(\lambda \right)d\lambda }{\int_{350\;nm}^{900\;nm}\phi_{ph}\left(\lambda \right)d\lambda }\times 100\%$$

where *EQE*(*λ*) is the external quantum efficiency at a given wavelength (*λ*) and *ϕ_ph_*(*λ*) is the photon flux of AM 1.5 G at that wavelength (*λ*). The ITO layer increased the EQE values between 450 nm and 900 nm wavelengths and layers of ITO/SiO_2_ increased the EQE values between 350 nm and 900 nm wavelengths, which were consistent with the obtained changes in optical reflectance. The overall *EQE_w_* of the GaAs solar cell with layers of ITO/SiO_2_ (77.15%) slightly exceeded that of the GaAs solar cell with an ITO layer (76.64%).

**Figure 9:**
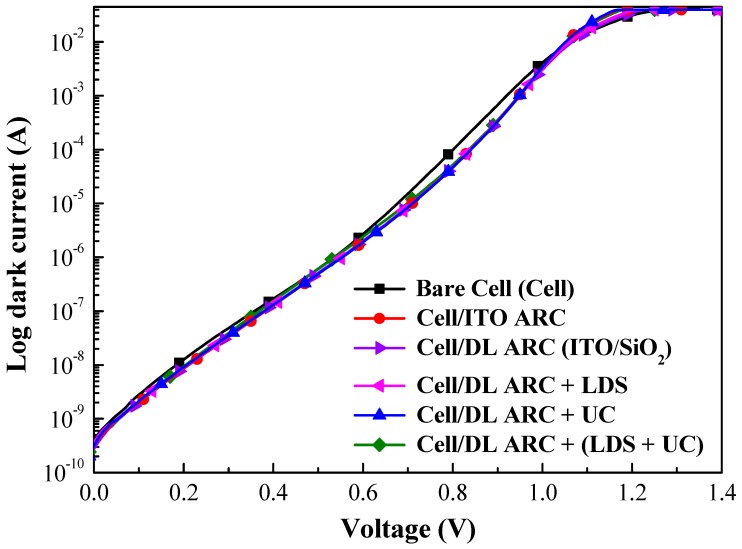
Dark I-V curves of a bare-type GaAs single-junction solar cell and GaAs solar cells coated with layer of ITO or ITO/SiO_2_ or SiO_2_ layer containing Eu-doped phosphor.

Figure 9 presents the dark I-V curves of a bare-type GaAs single-junction solar cell and GaAs solar cells coated with a layer of ITO or layers of ITO/SiO_2_, or GaAs solar cells coated with a SiO_2_ layer containing various phosphor particles. The ideality factor (*n*) and reverse saturation current density (*J*_0_) of the proposed GaAs solar cells are listed in Table 2. The *n* and *J*_0_ values were as follows: bare-type GaAs solar cell (2.12 and 5.04 × 10^−11^ A/cm^2^), ITO-coated GaAs solar cell (1.84 and 2.20 × 10^−12^ A/cm^2^), ITO/SiO_2_-coated GaAs solar cell (1.77 and 1.15 × 10^−12^ A/cm^2^), and the cells with a SiO_2_ layer containing Eu-doped phosphor particles (1.77 and 1.15 × 10^−12^ A/cm^2^). In this study, the passivation effects of the ITO layer, the ITO/SiO_2_ layer, or the SiO_2_ layer containing Eu-doped phosphor, on GaAs in suppressing surface recombination were found to be reduced for n and J_0_, as compared with that of the bare cells. In addition, the n and *J*_0_ values can be extracted from Log current versus voltage (Log I-V) curves by drawing an extended straight line along the I-V curves at the voltage between 0.8 V and 1.05 V, the intercept of the current axis by the straight line is the *J*_0_ value (or *I*_0_/*A*, A is active area of the diode) and the inverse slope of the straight line is proportional to the ideality factor, *n* [37].

**Figure 10:**
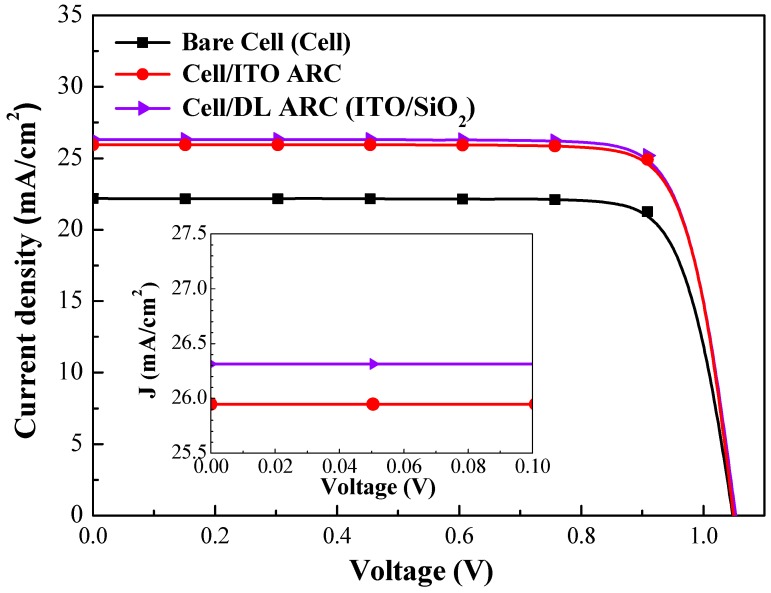
Photovoltaic J-V curves of a bare-type GaAs single-junction solar cell and cells coated with layer of ITO or ITO/SiO_2_.

Figure 10 presents the photovoltaic J-V curves of a bare-type GaAs single-junction solar cell and GaAs solar cells coated with a layer of ITO or layers of ITO/SiO_2_. The photovoltaic performance of the evaluated GaAs solar cells is summarized in Table 3. The short-circuit current density (*J_sc_*), open-circuit voltage (*V*_oc_), and conversion efficiency (*η*) of the bare-type GaAs single-junction solar cell were 22.19 mA/cm_2_, 1.047 V, and 19.33%, respectively. Compared to the bare-type cell, the short-circuit current density enhancement (Δ*J_sc_*) of 16.90% (from 22.19 to 25.94 mA/cm^2^) was obtained when the cell coated an ITO layer, whereas the coated ITO/SiO_2_ layers enhanced Δ*J_sc_* 18.52% (from 22.19 to 26.30 mA/cm^2^). The *J_sc_* improvement is in strong agreement with the *EQE_w_* enhanced values. In addition, the ITO layer enhanced conversion efficiency (Δ*η*) by 17.02% (from 19.33% to 22.62%), whereas the ITO/SiO_2_ layer enhanced Δ*η* by 18.52% (from 19.33% to 22.91%). We also made a comparison between measured *J_sc_* and *J_sc_* integrated from the EQE spectra (hereafter referred to as *J_SC-_*_EQE_) in Table 3. The *J*_*SC*-EQE_ values are almost the same as *J_sc_* values. Overall, the ITO/SiO_2_ layers outperformed the ITO layer in terms of enhanced optical and electrical performances (optical reflectance, EQE, and the dark I-V and photovoltaic J-V results) [32]. Thus, a layer configuration of ITO/SiO_2_ was applied to the GaAs solar cells tested in all subsequent experiments.

### 3.3. Optical and Electrical Performances of GaAs Single-Junction Solar Cells Coated with SiO_2_ Layer Containing Eu-Doped Phosphors, Yb/Er-Doped Phosphors, or A Combination of Eu-Doped and Yb/Er-Doped Phosphors

**Figure 11:**
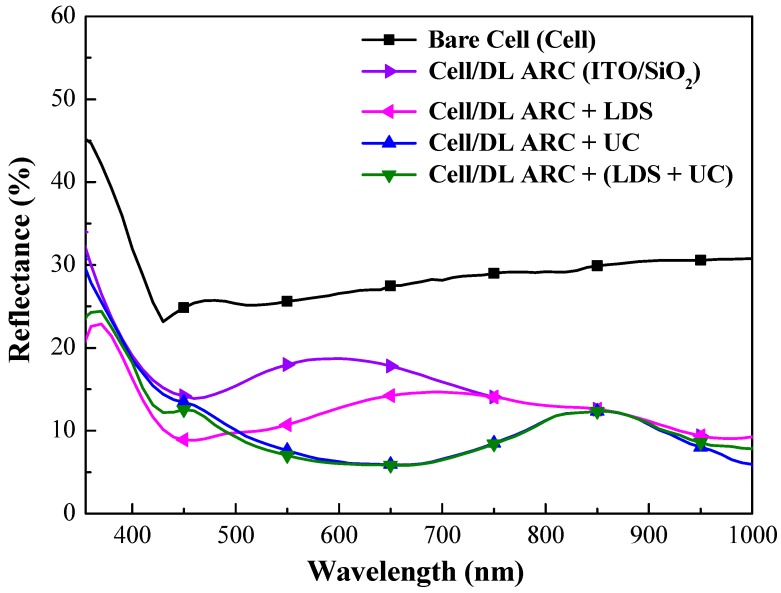
Optical reflectance spectra of bare-type GaAs single-junction solar cell (hereafter referred to as cell), cell/DL-ARC, cell/DL-ARC + LDS, cell/DL-ARC + UC, and cell/DL-ARC + (LDS + UC).

Figure 11 presents the optical reflectance spectra of a bare-type GaAs single-junction solar cell (hereafter referred to as cell), a GaAs solar cell with a double-layer antireflection coating of ITO/SiO_2_ (hereafter referred to as cell/DL-ARC), a GaAs solar cell with an SiO_2_ layer containing Eu-doped phosphors on the double-layer antireflection coating of ITO/SiO_2_ (hereafter referred to as cell/DL-ARC + LDS), a GaAs solar cell with an SiO_2_ layer containing Yb/Er-doped phosphors on the double-layer antireflection coating of ITO/SiO_2_ (hereafter referred to as cell/DL-ARC + UC), and a GaAs solar cell with an SiO_2_ layer containing Eu-doped as well as Yb/Er-doped phosphors on the double-layer antireflection coating of ITO/SiO_2_ (hereafter referred to as cell/DL-ARC + (LDS + UC)). Table 1 lists the *R_w_* values calculated for all evaluated GaAs cells. The optical reflectance of the cell with a DL-ARC displayed wideband antireflection characteristics (W-shapes) with the lowest reflectance at wavelengths of roughly 450 and 830 nm. This reflectance can be attributed to destructive interference in the light reflected at the air/SiO_2_, SiO_2_/ITO, and ITO/GaAs interfaces. The optical reflectance of the SiO_2_ layer containing phosphor particles was lower than the SiO_2_ layer without phosphor particles at wavelengths of 350 nm to 800 nm. The reduction in reflectance at 350 nm to 450 nm can be attributed to the absorption of incident light by Eu-doped phosphor particles (LDS effect). Note that the GaAs solar cell containing Eu-doped, as well as Yb/Er-doped phosphors, did not present a significant reduction in reflectance between 350 nm and 450 nm, due to the low concentration of Eu-doped in this configuration. In addition, the reduction in reflectance between 500 nm and 800 nm is attributed to the forward scattering of incident photons by both phosphor particles on the surface of the GaAs solar cell. Moreover, a slight reduction in reflectance between 950 nm and 1000 nm was attributed to the UC absorption by Yb/Er-doped phosphors.

**Figure 12:**
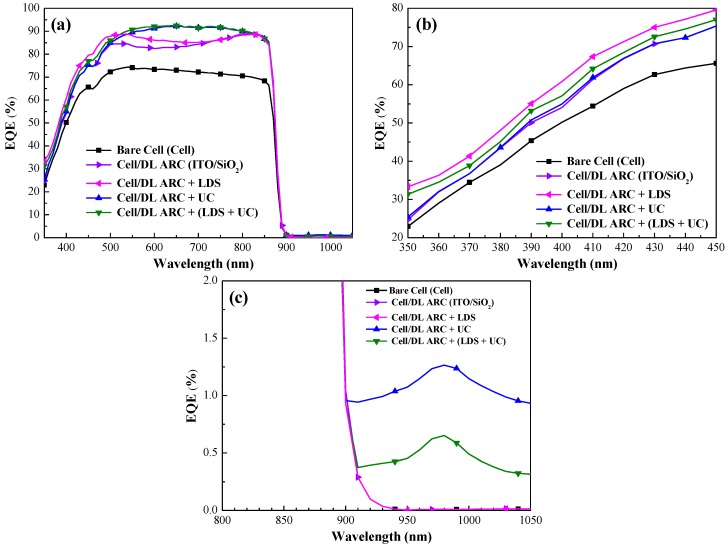
(**a**) EQE response of bare-type GaAs single-junction solar cell (cell), cell/DL-ARC (ITO/SiO_2_), cell/DL-ARC + LDS, cell/DL-ARC + UC, and cell/DL-ARC + (LDS + UC); (**b**) EQE response due to LDS effect at short wavelength region; and (**c**) EQE response due to UC effect at long wavelength region.

Figure 12a presents the EQE response of all GaAs single-junction solar cells evaluated from 350 nm to 1050 nm. Table 1 lists the *EQE_w_* values for all evaluated cells. The EQE values of the GaAs cell with DL-ARC were enhanced at wavelengths of 350 nm to 900 nm, due to the passivation and antireflection imposed by the ITO/SiO_2_ layers. This is in strong agreement with the optical reflectivity of the GaAs solar cell. The EQE values of the GaAs solar cell with a SiO_2_ layer containing Eu-doped phosphors (cell/DL-ARC + LDS) exceeded that of the GaAs solar cell with DL-ARC at wavelengths of 350 nm to 450 nm. This enhancement was attributed to the LDS-effects and forward scattering induced by the Eu-doped phosphor particles (Figure 12b). In addition, a weak EQE response can be observed at wavelengths 900 nm to 1050 nm, as shown in Figure 12c, due to the UC of Yb/Er-doped phosphor particles when those particles are excited by around 980 nm light (here absorbed 980 nm photon and re-emitted 650 nm to 670 nm photons into the cells). The EQE values of the GaAs solar cell with a SiO_2_ layer containing Yb/Er-doped phosphors were higher than that of the cell with DL-ARC at wavelengths between 500 nm and 850 nm, due to the UC-effects and forward-scattering induced by the Yb/Er-doped phosphor particles. The EQE response of the GaAs solar cell with a combination of Eu-doped (1.5 wt%) and Yb/Er-doped (1.5%) phosphor particles (cell/DL-ARC + (LDS + UC)) was nearly the same as the cell with 3 wt% UC phosphors (cell/DL-ARC + UC). This can be attributed to the fact that the UC photons (650–670 nm) absorbed in the GaAs had higher responsivity values than the LDS photons (518 nm) Thus, it would be reasonable to expect that an appropriate increase in the concentration of Eu-doped and Yb/Er-doped phosphor particles would further enhance the EQE response.

**Figure 13:**
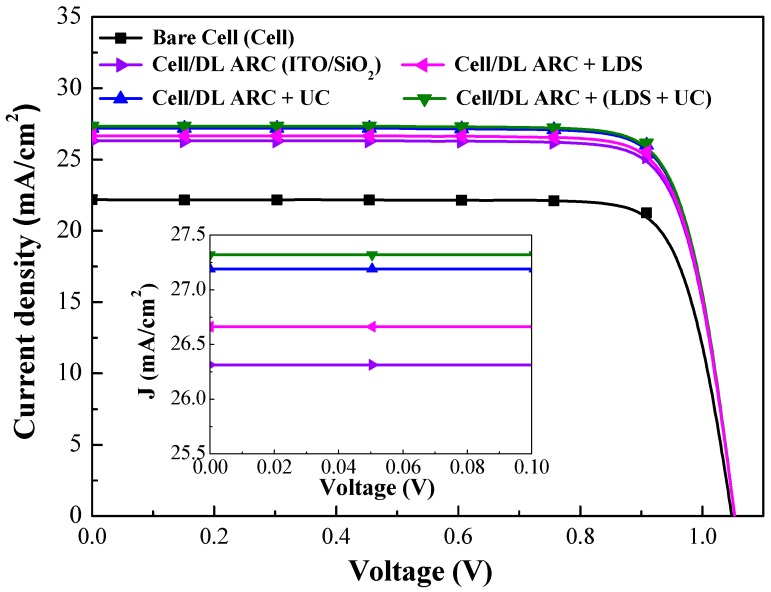
Photovoltaic J-V curves of bare-type GaAs single-junction solar cell (cell), cell/DL-ARC, cell/DL-ARC + LDS, cell/DL-ARC + UC, and cell/DL-ARC + (LDS + UC).

Figure 13 presents the photovoltaic J-V curves of GaAs single-junction solar cells evaluated in this study. Table 3 summarizes the photovoltaic performances of GaAs solar cells. The *J_sc_*, *V*_oc_, and *η* of the bare-type GaAs solar cell were 22.19 mA/cm^2^, 1.047 V and 19.33%, respectively. Compared to the bare-type cell, the cell/DL-ARC enhanced the *J_sc_* by 18.52% (from 22.19 to 26.30 mA/cm^2^) and *η* by 18.52% (from 19.33% to 22.91%), due to the antireflection and passivation effects. The inset of Figure 13 presents the photocurrent of the GaAs solar cell with DL-ARC and the GaAs solar cells with spectral conversion layers under near-zero voltage bias. These values clearly demonstrate the contribution of LDS (by Eu-doped phosphors) and UC (by Yb/Er-doped phosphors) in enhancing photocurrent. The *J_sc_* values of all GaAs solar cells with a spectral conversion layer (LDS layer or UC layer) exceeded that of the cell with only a DL-ARC. The *J_sc_* of the GaAs solar cell with a UC layer (3 wt% Yb/Er-doped phosphors) exceeded that of the GaAs solar cell with an LDS layer (3 wt% Eu-doped phosphors), due to the fact that UC photons are absorbed in the high responsivity regions of the GaAs cell. The *J_sc_* and *η* of the GaAs solar cell combining lower concentrations of Eu-doped (1.5 wt%) and Yb/Er-doped (1.5 wt%) phosphors exceeded that of the GaAs solar cells with higher concentrations of phosphors (3 wt%), due to the broadband emissions provided by LDS and UC photons. It is reasonable to expect that an appropriate increase in the combined concentration of Eu-doped and Yb/Er-doped phosphor particles (>1.5 wt%) would further enhance *J_sc_* and *η*.

## 4. Conclusions

The deposition of high-quality ITO film on GaAs solar cells via thermal sputtering was shown to reduce reflection and enhance passivation performance. The luminescent downshift provided by Eu-doped phosphors and the up-conversion provided by Yb/Er-doped phosphors, and forward scattering induced by phosphor particles were shown to enhance the conversion efficiency of GaAs single-junction solar cells. The SiO_2_ layer containing 3 wt% Yb/Er-doped phosphors improved the efficiency of GaAs solar cells by 22.71%, whereas the SiO_2_ layer containing 3 wt% Eu-doped phosphors improved efficiency by only 19.97%, due to the fact that UC photons were absorbed in the higher responsivity band of the GaAs solar cell. The conversion efficiency of the GaAs single-junction solar cell with a combination of 1.5 wt% Eu-doped phosphors and 1.5 wt% Yb/Er-doped phosphors (23.84%) exceeded that of the cells with 3 wt% Eu-doped or Yb/Er-doped phosphors (23.72%), due to the broadband emission provided by LDS and UC photons.

## Figures and Tables

**Table 1:** *R_w_* and *EQE_w_* of all evaluated cells.

GaAs Solar Cell	*R_w_* (%) (350–900 nm)	*EQE_w_* (%) (350–900 nm)
Bare cell (Cell)	27.91	64.84
Cell with/ITO ARC	15.04	76.64
Cell/DL-ARC (ITO/SiO_2_)	16.21	77.15
Cell/DL-ARC + LDS	12.65	79.61
Cell/DL-ARC + UC	10.10	80.97
Cell/DL-ARC + (LDS + UC)	9.75	81.49

**Table 2:** Ideality factor (*n*) and saturation current density (*J*_0_) of bare-type GaAs solar cell and GaAs solar cells coated with ITO, ITO/SiO_2_ layer, or SiO_2_ layer containing Eu-doped phosphor.

	Ideality Factor (*n*)	Saturation Current (*J*_0_) A/cm^2^
Bare cell (cell)	2.12	5.04 × 10^−11^
Cell/ITO ARC	1.84	2.20 × 10^−12^
Cell/DL-ARC (ITO/SiO_2_)	1.77	1.15 × 10^−12^
Cell/DL-ARC + LDS	1.77	1.15 × 10^−12^
Cell/DL-ARC + UC	1.77	1.02 × 10^−12^
Cell/DL-ARC + (LDS + UC)	1.79	1.40 × 10^−12^

**Table 3:** Photovoltaic performances of all evaluated cells under AM 1.5 G solar simulations.

GaAs Solar Cell	V_oc_ (V)	*J_sc_* (mA/cm^2^)	*J*_sc-EQE_ (mA/cm^2^)	F.F. (%)	*η* (%)	△*J_sc_* (%)	△*η* (%)
Bare cell (cell)	1.0468	22.19	21.92	83.21	19.33	---	---
Cell/SiO_2_ ARC	1.0495	25.53	25.44	82.61	22.13	15.05	14.48
Cell with/ITO ARC	1.0542	25.94	25.79	82.75	22.62	16.90	17.02
Cell with/DL-ARC (ITO/SiO_2_)	1.0528	26.30	25.91	82.74	22.91	18.52	18.52
Cell with/DL-ARC + LDS	1.0531	26.65	26.48	82.63	23.19	20.10	19.97
Cell with/DL-ARC + UC	1.0537	27.19	27.12	82.79	23.72	22.53	22.71
Cell with/DL-ARC + (LDS + UC)	1.0537	27.33	27.32	82.79	23.84	23.16	23.33
